# Engineered CRISPR/Cas9 System for Transcriptional Gene Silencing in *Arthrobacter* Species Indicates Bacterioruberin is Indispensable for Growth at Low Temperatures

**DOI:** 10.1007/s00284-022-02887-5

**Published:** 2022-05-20

**Authors:** Alexander Flegler, André Lipski

**Affiliations:** Institute of Nutritional and Food Science, Food Microbiology and Hygiene, University of Bonn, Friedrich-Hirzebruch-Allee 7, 53115 Bonn, Germany

## Abstract

Pink-pigmented *Arthrobacter* species produce the rare C_50_ carotenoid bacterioruberin, which is suspected to be part of the cold adaptation mechanism. In silico analysis of the repertoire of genes encoded by the *Arthrobacter agilis* and *Arthrobacter bussei* genome revealed the biosynthetic pathway of bacterioruberin. Although genetic analysis is an essential tool for studying the physiology of *Arthrobacter* species, genetic manipulation of *Arthrobacter* is always time and labor intensive due to the lack of genetic engineering tools. Here we report the construction and application of a CRISPR/deadCas9 system (pCasiART) for gene silencing in *Arthrobacter* species. The engineered system pCasiART is suitable for the Golden Gate assembly of spacers, enabling rapid and accurate construction of adapted systems. In addition, pCasiART has been developed to provide an efficient transcription inhibition system for genome-wide gene silencing. The gene silencing of the phytoene synthase (CrtB), the first enzyme in bacterioruberin biosynthesis, suppressed bacterioruberin biosynthesis in *Arthrobacter agilis* and *Arthrobacter bussei*, resulting in a lack of pink pigmentation, reduction of biomass production, and growth rates at low temperatures.

**Supplementary Information:**

The online version contains supplementary material available at 10.1007/s00284-022-02887-5.

## Introduction

Carotenoids are among the most diverse natural pigments found in animals, plants, fungi, bacteria, and archaea. Currently, the Carotenoids Database provides information on 1204 natural carotenoids in 722 source organisms [1]. The biosynthesis pathway from isopentenyl pyrophosphate (IPP) and dimethylallyl pyrophosphate (DMAPP) to phytoene is present in most carotenoid-producing bacteria. IPP and its isomer DMAPP are the primary building blocks of carotenoids produced by the mevalonate and the 2-*C*-methyl-d-erythritol 4-phosphate (MEP) pathways in bacteria. Most carotenoids consist of long-chain polyenes synthesized by condensing two C_20_ geranylgeranyl pyrophosphate (GGPP) to phytoene using the phytoene synthase (CrtB). The transformation of phytoene by desaturation, isomerization, cyclization, and other modifications leads to the production of various carotenoids [2]. Although numerous bacteria produce carotenoids and their genomes encode carotenoid biosynthetic pathways, only < 10% of these pathways have been experimentally validated [3].

The genus *Arthrobacter*, described by Conn and Dimmick [4] and amended by Busse [5], is a common group of bacteria isolated from various sources such as soil, air, food, water, and plants that can produce a wide variety of pigments of different colors, e.g., yellow, red, green, and blue [6]. The C_50_ carotenoid bacterioruberin and its glycosylated derivatives are expected to play a crucial role in the cold adaptation of pink-pigmented *Arthrobacter* species, as shown by supplementation experiments [7, 8]. The rarely occurring bacterioruberin is derived from the C_40_ structure by adding two C_5_ isoprene units, which may be modified by further desaturation and hydroxylation [9–11]. In addition, the production of the acyclic C_50_ carotenoid bacterioruberin is typical in extremely halophilic archaea and psychrophilic bacteria [6]. Although some *Arthrobacter* species produce bacterioruberin, the biosynthetic pathway in these bacteria has not been elucidated. In addition to that, only a few *Arthrobacter* species have been genetically modified and were studied due to the few existing genetic engineering tools. Nevertheless, although transposon mutagenesis systems and homologous recombination systems have already been developed and performed to create mutants of *Arthrobacter* strains, genetic manipulation is always time consuming and labor intensive [12]. For example, knocking out a gene via homologous recombination requires typically two separated crossover steps. First, the editing plasmid is integrated into the target locus by homologous recombination, which is achieved by incubating the cells at a nonpermissive temperature. Second, removal of the integrated plasmid is promoted by growing the cells at a permissive temperature, and loss of the editing plasmid in the genome is facilitated by a counter-selection method. As a result, the entire genome editing takes a minimum of one week. The discovery of the CRISPR/Cas9 system provides a simple, sequence-specific platform to generate a double-stranded DNA break in the target genome, making it possible to select double-crossing events in one step [13]. A previous study by Chen et al. [14] demonstrated that CRISPR/Cas9 was successfully used for genome editing in *Staphylococcus aureus*. Additionally, by converting the active sites, Asp_10_ and His_840_ to Ala of the Cas9 protein, they constructed the highly efficient transcription inhibition system pCasiSA. One significant advantage of using the CRISPR/Cas9 system for gene silencing is the fast and easy assembly of the genome-targeting module, the spacer, compared with other tools [14]. In addition, a pool of spacers can be readily synthesized using the high-throughput DNA synthesis technique. Using the Golden Gate assembly, it is possible to simultaneously assemble a collection of spacers, enabling rapid and accurate construction of a library for genome-wide studies using the catalytically inactive dead Cas9 protein (Casi9).

Here we decipher in silico the biosynthetic pathway of bacterioruberin of *Arthrobacter* species. Furthermore, we report the construction and application of a CRISPR/deadCas9 system (pCasiART) for gene silencing in *Arthrobacter* species. The engineered system pCasiART is suitable for efficient transcription inhibition and gene silencing. The system was applied here for gene silencing *crtB*, producing the first enzyme in bacterioruberin biosynthesis to suppress bacterioruberin biosynthesis. With this approach, we demonstrated the role of bacterioruberin for growth under low-temperature conditions for pink-pigmented *Arthrobacter* strains.

## Materials and Methods

### Bacterial Strains, Plasmids, and Culture Conditions

The bacterial strains and plasmids used in this study are listed in Table S1. Plasmids pART2, pART2-*gfp*, and pCasiSA were gratefully provided by Prof. Susanne Fetzner (University of Münster, Germany) and Prof. Dr. Quanjiang Ji (ShanghaiTech University, China), respectively. NEB 5-alpha-Competent *Escherichia coli* (New England Biolabs, UK) cells were used for the molecular cloning procedure and grown in lysogeny broth (LB) or on LB agar plates. Two *Arthrobacter* strains of two different species were examined. Both species belong to the “Pink *Arthrobacter agilis* group” within the “*Arthrobacter agilis* group,” showing a more intense pigmentation at low growth temperatures [15]. *Arthrobacter agilis* DSM 20550^T^ and *Arthrobacter bussei* DSM 109896^T^ were aerobically cultured in 100 ml tryptic soy broth (TSB) containing 17 g peptone from casein L^−1^, 3 g peptone from soy L^−1^, 2.5 g d-glucose L^−1^, 5 g sodium chloride L^−1^, and 2.5 g dipotassium hydrogen phosphate L^−1^ using 300-ml Erlenmeyer flasks or on tryptic soy agar (TSA) plates. Growth in the TSB medium was documented by optical density (OD) at 625 nm with a GENESYS 30 visible spectrophotometer (Thermo Fisher Scientific, USA). Cultures were prepared in independent replicates, inoculated with 1% (vol/vol) of overnight culture, and incubated on an orbital shaker at 10 or 30 °C and 150 rpm in the dark until the late exponential phase (OD_625_ = 1–1.2). When appropriate, kanamycin was added to the medium at final concentrations of 140 μg ml^−1^ for *A*. *agilis* or *A*. *bussei* strains after electroporation and 30 μg ml^−1^ for *E. coli* after transformation.

### Construction of pCasiART

Standard DNA manipulation and cloning methods were used [16]. Plasmid Miniprep Kit, DNA Gel Extraction Kit, restriction enzymes, T4 ligase, Q5 High-Fidelity 2X Master Mix, and Q5 Site-Directed Mutagenesis Kit were obtained from New England Biolabs (Ipswich, UK) and used according to the manufacturer’s instructions. Oligonucleotides and synthesized gBlock were obtained from Eurofins MWG (Ebersberg, Germany). Primers used in this study are listed in Table S2. The pCasiART plasmid was constructed using the following procedures: The gene encoding the catalytically inactive Cas9 was amplified from the pCasiSA plasmid [14] and inserted into the *Sal*I/*Avr*II sites of the pART2 plasmid [17]. Next, the *hdnO* promoter and the sgRNA fragment were synthesized as a gBlock and inserted into the *Bsu36*I/*Avr*II sites of the previously generated plasmid. Afterward, a single-base substitution a_7320_ to g_7320_ was performed to eliminate the hindering *Bsa*I site in the origin of replication *pCG100* for subsequent spacer insertion. Additionally, *lacZ*α, *lac* operator, and *lac* promoter were amplified from the pUC19 plasmid [18] and inserted into the *Bsa*I/*Bsa*I sites for the blue–white screen of the successfully integrated spacer, resulting in the final pCasiART plasmid. The success of constructing the pCasiART plasmid was verified by PCR, enzyme digestion, and sequencing. Designing the spacers of interest for pCasiART (pCasiART-spacer) with related Golden Gate assembly is explained in detail in the supplementary information. The detailed cloning history of pCasiART is shown in Fig. S1.

## Results and Discussion

### Analysis of Genes of the Bacterioruberin Biosynthetic Pathway in the Genomes of *Arthrobacter agilis *and *Arthrobacter bussei*

**Fig. 1:**
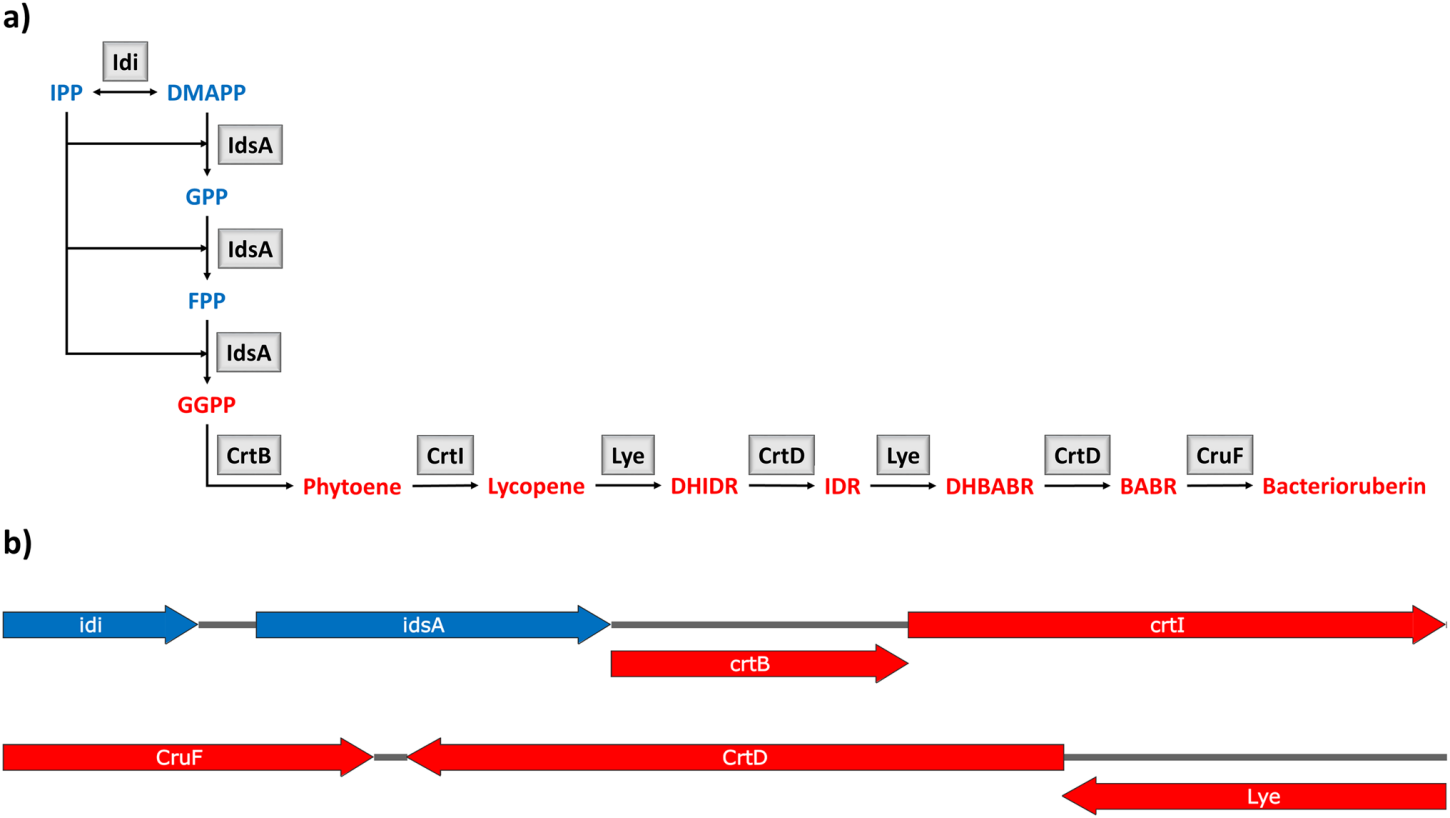
**a** Bacterioruberin biosynthesis pathway of *A*. *agilis* and *A*. *bussei* and **b** genetic organization of bacterioruberin genes. Intermediates from the 2-*C*-methyl-d-erythritol 4-phosphate (blue) and the bacterioruberin pathway (red) are isopentenyl pyrophosphate (IPP), dimethylallyl pyrophosphate (DMAPP), farnesyl pyrophosphate (FFP), geranylgeranyl pyrophosphate (GGPP), phytoene, lycopene, dihydroisopentenyldehydrorhodopin (DHIDR), isopentenyldehydrorhodopin (IDR), dihydrobisanhydrobacterioruberin (DHBABR), bisanhydrobacterioruberin (BABR), and bacterioruberin. Enzymes involved in different steps of the pathway are isopentenyl pyrophosphate isomerase (Idi), geranylgeranyl pyrophosphate synthase (IdsA), phytoene synthase (CrtB), phytoene desaturase (CrtI), lycopene elongase/hydratase (Lye), carotenoid-3,4-desaturase (CrtD), and bisanhydrobacterioruberin hydratase (CruF) (Color figure online)

The present study revealed in silico the biosynthetic pathway of the carotenoids present in *A*. *agilis* and *A*. *bussei* based on bioinformatics data (Fig. 1a). Function and pathway analyses were performed using the BlastKOALA web tool of the Kyoto Encyclopedia of Genes and Genomes (KEGG) database [19]. Genome analysis of *A*. *agilis* strain NCTC2676_1 (GCF_900631605.1) and *A*. *bussei* strain DSM 109896^T^ (GCF_009377195.2) revealed that both have all genes involved in the MEP pathway [20] for the synthesis of geranyl pyrophosphate from the isoprenoid precursors IPP, DMAPP, and other carotenoid biosynthetic genes. The MEP pathway genes annotated in *A*. *agilis* are *dxs* (WP_087026194.1), *dxr* (WP_087030629.1), *ispD* (WP_087028001.1), *ispE* (WP_087030977.1), *ispF* (WP_087028003.1), *ispG/gcpE* (WP_087030618.1), *ispH* (WP_087029689.1), *idi* (WP_087030401.1), and two homologs of *idsA* (WP_087025706.1, WP_087030119.1). The MEP pathway genes annotated in *A*. *bussei* DSM 109896^T^ are *dxs* (WP_152814802.1), *dxr* (WP_152816690.1), *ispD* (WP_152816505.1), *ispE* (WP_152815970.1), *ispF* (WP_152816409.1), *ispG/gcpE* (WP_055772581.1), *ispH* (WP_152812126.1), *idi* (WP_152812423.1), and two homologs of *idsA* (WP_152814964.1, WP_152812421.1).

The functional analysis with BlastKOALA of coding DNA sequence (CDS) revealed genes for potential carotenoid biosynthesis in *A*. *agilis* and *A*. *bussei*. However, this analysis annotated only two genes encoding for bacterioruberin biosynthesis. Additional homology analysis, using standard protein BLAST with non-redundant UniProtKB/SwissProt sequences [21], revealed candidate genes encoding enzymes for bacterioruberin biosynthesis. WP_158250107.1 (*A*. *agilis*) and WP_152813953.1 (*A*. *bussei*) are annotated as squalene/phytoene synthase family protein and had 35% and 36.1% amino acid sequence identity to CrtB of *Streptomyces griseus* and 26.3% and 26.8%, respectively, with that of *Pantoea agglomerans*. Furthermore, WP_179195224.1 (*A*. *agilis*) and WP_152812419.1 (*A*. *bussei*) are annotated as phytoene desaturase (CrtI) and had 43.1% and 42.4% amino acid identity with that of *Haloarcula japonica* strain DSM 6131 and 28.7% and 29.1%, respectively, from that of *Staphylococcus aureus* subsp. *aureus* MRSA252. The genes *idi*, *idsA*, *crtB*, and *crtI* cluster in that order on the genome of *A*. *agilis* and *A*. *bussei* (Fig. 1b). It is known that the genes related to carotenoid synthesis are arranged in clusters or neighborhoods in some bacteria [22, 23]. Based on the pathway annotation using KEGG Database, CrtI was involved in the multistep conversion of phytoene into lycopene. In addition, in the bacterioruberin biosynthetic pathway, lycopene is used as a precursor and converted to bacterioruberin by introducing two C_5_ isoprene units, two double bonds, and four hydroxyl groups into lycopene. Pink-pigmented *Arthrobacter* species produce bacterioruberin-type carotenoids [8, 15], but the complete pathway for the biosynthesis of bacterioruberin in pink-pigmented *Arthrobacter* species is not yet known. Some coding sequences (CDS) were predicted to be candidate genes encoding enzymes for the biosynthesis of bacterioruberin. For example, lycopene elongase (Lye) catalyzes the committed step in bacterioruberin biosynthesis [11]. Lye converts lycopene into dihydroisopentenyldehydrorhodopin (DHIDR). In the genome of *A*. *agilis* and A*. bussei*, WP_087030409.1 and WP_152813957.1 are annotated as UbiA family prenyltransferase showed 37.9% and 38.3% amino acid identity with Lye from *Dietzia* sp. strain CQ4 and 36.8% and 36.3%, respectively, of *Halobacterium salinarum* strain NRC-1. 1-Hydroxy-2-isopentenylcarotenoid 3,4-desaturase (CrtD) further converts DHIDR into isopentenyldehydrorhodopin (IDR), which is converted to dihydrobisanhydrobacterioruberin (DHBABR) by Lye. DHBABR is converted to bisanhydrobacterioruberin (BABR) by CrtD. Homology analysis revealed that WP_087030149.1 of *A*. *agilis* and WP_152812439.1 of *A*. *bussei* are annotated as a phytoene dehydrogenase-related protein had 27.4% and 28.7%, respectively, amino acid identity with CrtD from *Haloarcula japonica*. Bisanhydrobacterioruberin hydratase (CruF) is responsible for the final conversion of BABR into BR in various halophilic bacteria. The protein WP_158250106.1 of *A*. *agilis* and WP_191931588.1 of *A*. *bussei* are annotated as a carotenoid biosynthesis protein with 44.1% and 42.3%, respectively, amino acid identity with CruF from *Haloarcula japonica* strain DSM 6131. The genes of Lye, CrtD, and CruF cluster in that order in the genome of *A*. *agilis* and *A*. *bussei* (Fig. 1b). Homologs of the carotenoid 1,2-hydratase (CrtC), involved in the spirilloxanthin biosynthetic pathway, could not be found in the genome of *A*. *agilis* and *A*. *bussei*. Some of the products predicted from this synthesis pathway, bisanhydrobacterioruberin and bacterioruberin, have already been detected in a previous report and supported the presence and activity of the enzymes predicted from the genome information [15].

### Engineering a CRISPR/Cas9 System for Gene Silencing in *Arthrobacter* Species

**Fig. 2:**
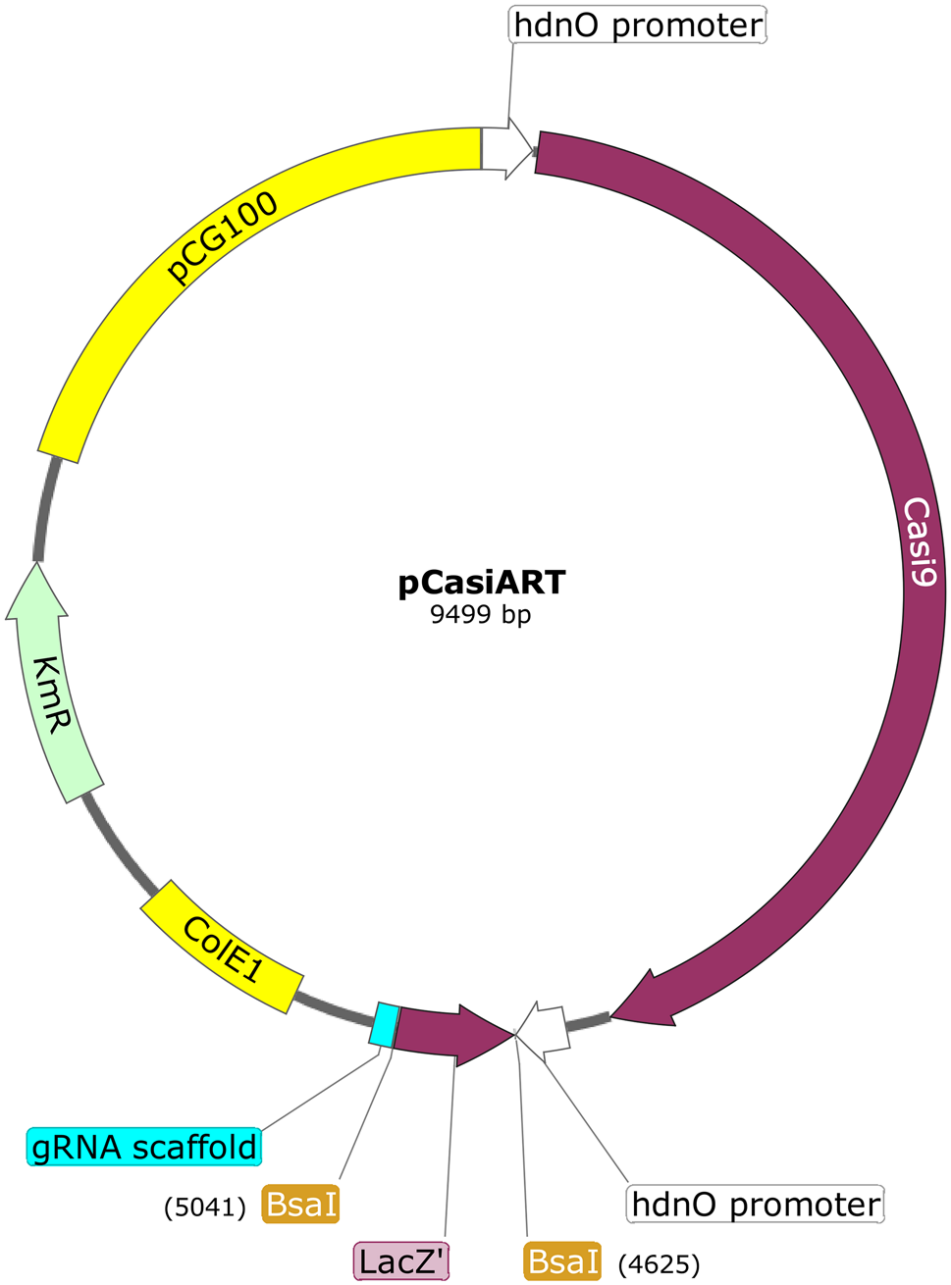
Annotated plasmid map of pCasiART. *hdnOp*, promoter of the 6-D-hydroxynicotine oxidase gene (*hdnO*) from plasmid pAO1 used for expression of catalytic inactive dead Cas9 (Casi9) and gRNA scaffold; Casi9, Cas9 protein from *Streptococcus pyogenes* with mutation of Asp_10_ and His_840_ to Ala; *Bsa*I sites for Golden Gate assembly of the spacer; *lacZ*α, a fragment of β-galactosidase for the blue–white screen, spacer insertion site; KmR, the kanamycin-resistant marker in *E*. *coli* and *Arthrobacter* sp.; ColE1, a replication origin for *E*. *coli*; pCG100, a cryptic fragment from *Corynebacterium glutamicum* ATCC 13058 that can replicate autonomously in *Arthrobacter* species. Created with SnapGene® software (Insightful Science; available at snapgene.com). (Color figure online)

As demonstrated by the success of Casi9 for transcriptional inhibition in *S*. *aureus* [14], we designed and constructed the transcription inhibition system pCasiART for use with *Arthrobacter* sp. (Fig. 2). Following the approach of Chen et al. [14], we developed the analogous system pCasiART, with pART2 [17] as the backbone, an effective tool for fast and accurate screening of genes and pathways of interest in *Arthrobacter* species. The detailed functionality of this CRISPR/deadCas9 system has been described previously [14]. Therefore, we adapted the available *S*. *aureus* transcription inhibition system pCasiSA using the catalytically inactive dead Cas9 protein (Casi9) in *Arthrobacter* strains. An advantageous feature of this system is the presence of two seamless cloning sites. The *Bsa*I sites are used for one-step assembly of spacers by golden gate assembly, and the *Xba*I and *Xho*I sites are used for one-step Gibson assembly-mediated cloning of repair arms for homologous recombination-mediated repair after a DNA double-strand break [24]. Furthermore, the plasmid pCasSA contains the gene for the well-studied Cas9 protein from *Streptococcus pyogenes* [25, 26], in which its expression is driven by a strong *rpsL* promoter from *S*. *aureus*. On the other hand, the transcription of sgRNA is driven by the strong promoter *cap 1A*. Because the *rpsL* and *cap 1A* promoter in *A. agilis and A. bussei* has not been detected, we replaced both promoters with the well-studied strong promoter/operator of the 6-d-hydroxynicotine oxidase gene (*hdnO*) from *Arthrobacter oxidans* [27] to drive the expression of Casi9 and sgRNA. The functionality of pART2 was tested in advance with pART2-*gfp* in *A. agilis* DSM 20550^ T^ and *A*. *bussei* DSM 109896^ T^. Both strains produced bright fluorescence when electroporated with pART2-*gfp*, confirming the functionality of the *hdnO* promoter for the mentioned purpose.

### Silencing *crtB *Reveals that Bacterioruberin is Indispensable for Growth at Low Temperatures in Pink-Pigmented *Arthrobacter* Species

**Fig. 3:**
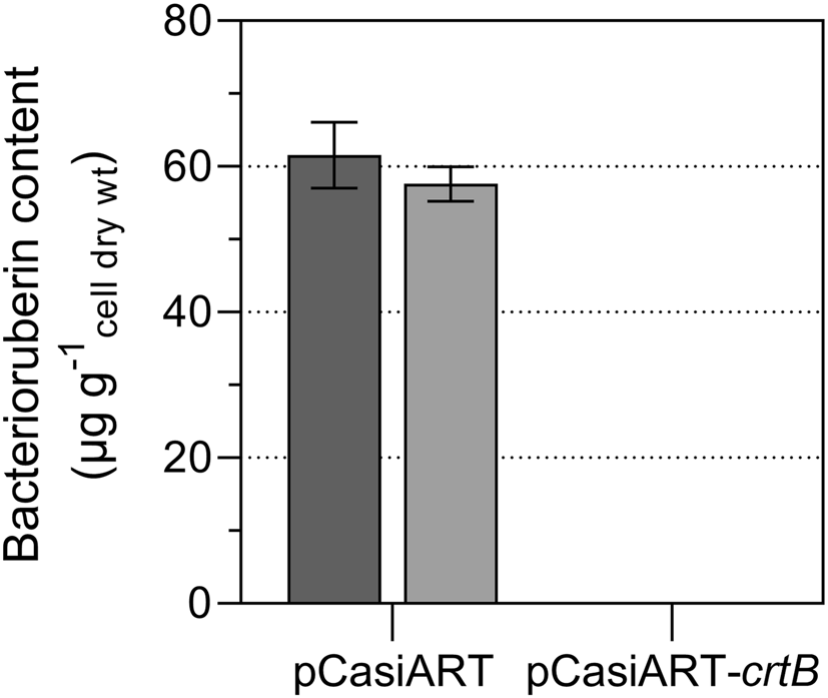
Bacterioruberin content after CRISPR/Cas9-mediated transcription inhibition of *crtB*. Total bacterioruberin content of strains *Arthrobacter agilis* DSM 20550^T^ (dark gray) and *Arthrobacter bussei* DSM 109896^T^ (gray) grown at 20 °C. Values are means ± standard deviation (*n* = 3)

Bacterioruberin is assumed to support cold adaptation in pink-pigmented *Arthrobacter* species, as indicated by suppression experiments [7]. Therefore, specific silencing of bacterioruberin synthesis is indispensable to demonstrate the impact of bacterioruberin on cold adaptation and to detect other phenotypes associated with the synthesis of bacterioruberin. So far, there has been no molecular suppression of the bacterioruberin content. Therefore, we silenced the *crtB* gene of *A. agilis* DSM 20550^T^ and *A*. *bussei* DSM 109896^T^ with pCasiART to disrupt the bacterioruberin production. The efficiency of this gene silencing system was evaluated by comparing the bacterioruberin content in cells carrying the empty pCasiART plasmid or the pCasiART plasmid with the *crtB* spacer (pCasiART-*crtB*). The total bacterioruberin was extracted and quantified as described previously [28, 29]. As shown in Fig. 3, the bacterioruberin content in both strains was drastically reduced and below the detection limit of 0.2 µg cell dry weight g^−1^ after introducing the spacers, demonstrating the effective transcriptional inhibition caused by the pCasiART system. In addition, colony formation and growth at 20 °C of both strains carrying pCasiART-*crtB* were slower than those carrying pCasiART. At a growth temperature of 10 °C, colony formation of both strains with pCasiART-*crtB* was absent. Thus, these results demonstrate the importance of bacterioruberin biosynthesis at decreasing growth temperature for pink-pigmented *Arthrobacter* strains. After curing the plasmid on TSA plates without kanamycin, we observed that both strains regained their typical pink pigmentation. The experiments were not performed at 30 °C because both species grow very slowly with pCasiART or pART2-*gfp* on TSA with kanamycin at this temperature.

## Conclusion

The development of the pCasiART system would enable accurate genome wide and defined screening of gene libraries, which cannot be achieved with conventional screening tools, such as transposon-mediated screening in *Arthrobacter* sp. We developed a highly efficient CRISPR/deadCas9-mediated transcriptional inhibition system for *Arthrobacter* sp., enabling fast and accurate screening of genes and pathways responsible for the phenotypes of interest. Furthermore, these results report the first gene silencing in *Arthrobacter* species by a CRISPR/Cas9 system. Introducing modern DNA assembly techniques into the system would significantly reduce the time and effort required. Further use and optimizations of the pCasiART system should dramatically accelerate various studies in *Arthrobacter* sp. and related bacteria, such as enzymology, natural product extraction, gene characterization, and other basic science research in microbiology as well as interdisciplinary research in chemical biology and synthetic biology.

## Supplementary Information

Below is the link to the electronic supplementary material.

Supplementary file1 (PDF 632 kb)

## Data Availability

The datasets/materials generated and analyzed during the current study are available on request from the corresponding author.
