# Supplementary material for: Engineered CRISPR/Cas9 System for Transcriptional Gene Silencing in Arthrobacter Species Indicates Bacterioruberin is Indispensable for Growth at Low Temperatures
Source: Curr Microbiol. 2022 May 20;79(7):199. doi: 10.1007/s00284-022-02887-5 (PMC9122864; doi:10.1007/s00284-022-02887-5)
Supplement: Supplementary file 1 — Supplementary file1 (PDF 632 kb) [file 284_2022_2887_MOESM1_ESM.pdf]

Supplementary Information

Engineered CRISPR/Cas9 System for Transcriptional Gene Silencing in *Arthrobacter* Species Indicates Bacterioruberin is Indispensable for Growth at Low Temperatures

Alexander Flegler[1] and André Lipski[1]*

[1]Institute of Nutritional and Food Science, Food Microbiology and Hygiene, University of Bonn, Friedrich-Hirzebruch-Allee 7, 53115 Bonn, Germany

***Correspondence**

e-mail: lipski@uni-bonn.de

This document contains supplementary information, including detailed protocol concerning designing spacers of interest for pCasiART and the construction history of pCasiART (Fig. S1).

DETAILED PROTOCOL

The pCasiART plasmids for transcriptional gene inhibition were assembled using the following protocol:

1. **Oligo Design**

   A 20 bp-spacer sequence was selected before 5'-NGG-3' (5'-NGG-3' was not included in the spacer) of the target gene. A ratio of 40~60% GC is the best. The two oligos were synthesized in the following form:

   Oligo I: 5'- GAAANNNNNNNNNNNNNNNNNNNN -3'

   Oligo II: 3'-NNNNNNNNNNNNNNNNNNNNCAAA -5'

2. **Phosphorylation**

2 µl	Oligo **I** (50 µM)
2 µl	Oligo **II** (50 µM)
5 µl	T4 DNA Ligase Buffer (10X) (NEB)
1 µl	T4 Polynucleotide Kinase (NEB)
40 µl	Nuclease-free H_2O
50 µl	

   Incubated at 37°C for 1 hour.

3. **Annealing**

   2.5 µl of 1 M NaCl was added to the phosphorylated oligo pairs, incubated at 95 °C for 3 min, and slowly cooled down to room temperature using a thermocycler. Next, the annealed oligos were diluted 20 times using Nuclease-free H_2O.

4. **Golden Gate assembly**

xx µl	20 fmol pCasi9ART plasmid
1 µl	diluted annealed oligos (100 fmol)
0.5 µl	T4 DNA Ligase Buffer (10X) (NEB)
0.5 µl	T4 Polynucleotide Kinase (NEB)
xx µl	Nuclease-free H_2O
10 µl	

37 °C	2 min	25 cycles
16 °C	5 min	
50 °C	5 min	
80 °C	15 min	
10 °C	infinite	

5. **Transformation**

   10 µl product of Golden Gate assembly was transformed into 100 µl 5-alpha Competent *E. coli* cells (NEB). White colonies were selected via blue/white screening on an LB agar plate containing 80 µg X-gal ml^{-1}, 0.3 mM IPTG, and 30 µg kanamycin ml^{-1}. The success of constructing the pCasiART-spacer plasmid was verified by PCR or sequencing.

Tables

Table S1 Bacterial strains and plasmids used in this study.

Strains and plasmids	Relevant genotype and description or sequence	Reference or source
**Plasmids**		
pUC19	Ap^R, *lacZ'*, *ColE1* ori, MCS	[1]
pART2	Km^R, *hdnO* promoter, *pCG100* ori, *ColE1* ori, MCS, His_8-tag	[2]
pART2-*gfp*	Km^R, *hdnO* promoter, *gfp*, *pCG100* and *ColE1* ori, MCS, His_8-tag	[2]
pCasiSA	Km^R, Cm^R, *repF* ori, *ColE1* ori, *cap 1A* promoter, *rpsL* promoter, sgRNA, dead Cas9	[3]
pEX-K168_gblock	Synthesized gBlock with *Bsa*I sites containing *hdnO* promoter along with the sgRNA fragment, Km^R, *ColE1* ori	This study
pART2-Casi9	*Sal*I/*Avr*II fragment of PCR-amplified dead Cas9 from pCasiSA in Sali/AvrII of pART2	This study
pART2-Casi9gBlock	*Avr*II/*Bsu36*I fragment containing *hndo* promoter and sgRNA from pEX-K168_gblock in *Avr*II/*Bsu36*I of pART2-Casi9	This study
pART2-Casi9gBlockori*	Substitution in pCG100 ori a_{7320} to g_{7320} of pART2-Casi9gBlock	This study
pCasiART	*Bsa*I fragment of PCR-amplified *lacZ'*, from pUC19 in *Bsa*I of pART2-Casi9gBlockori*	This study
pCasiART-*crtB*	pCasiART with *crtB* spacer in *Bsa*I/*Bsa*I	This study
**Strains**		
E. coli NEB 5-alpha	*fhuA2Δ(argF-lacZ)U169 phoA glnV44 Φ80 Δ(lacZ)M15 gyrA96 recA1 relA1 endA1 thi-1 hsdR17*	New England Biolabs
Arthrobacter agilis DSM 20550^T	Wild type	[4]
A. agilis pART2-*gfp*	Km^R, *A. agilis* DSM 20550^T carrying pART2-*gfp*	This study
A. agilis pCasiART	Km^R, *A. agilis* DSM 20550^T carrying pCasiART	This study
A. agilis pCasiART-*crtB*	Km^R, *A. agilis* DSM 20550^T carrying pCasiART-*crtB*	This study
Arthrobacter bussei DSM 109896^T	Wild type	[5]
A.bussei pART2-*gfp*	Km^R, *A. bussei* DSM 109896^T carrying pART2-*gfp*	This study
A. bussei pCasi	Km^R, *A. bussei* DSM 109896^T carrying pCasiART	This study
A. bussei pCasi-*crtB*	Km^R, *A. bussei* DSM 109896^T carrying pCasiART-*crtB*	This study

Table S2 Primers used to construct pCasiART and pCasiART-*crtB*.

Name	Sequence (5'-3')	Description
Casi9_F	ATTAGTCGACatggataagaaatactcaataggc (*Sal*I)	amplification of *cas9* from pCasiSA
Casi9_R	TAATCCTAGGttcaccgtcatcaccgaaac (*Avr*II)	amplification of *cas9* from pCasiSA
Q5SDM_a7320g_F	gccaagagagGgaccctacgg	eliminate *Bsa*I site in *pCG100*
Q5SDM_a7320g_R	ctcctttctaggtcgggc	eliminate *Bsa*I site in *pCG100*
LacZ_F	GGAAATGAGACCctatgcggcatcagagcagattgta (*Bsa*I)	amplification of the *lacZ*' from pUC19 plasmid
LacZ_R	AAAACTGAGACCtttacactttatgcttccggctc (*Bsa*I)	amplification of the *lacZ*' from pUC19 plasmid
crtBspacer_F	GAAACGAGTAGCAGCGGATGACCT	spacer for gene silencing *crtB*
crtBspacer_R	AAACAGGTCATCCGCTGCTACTCG	spacer for gene silencing *crtB*

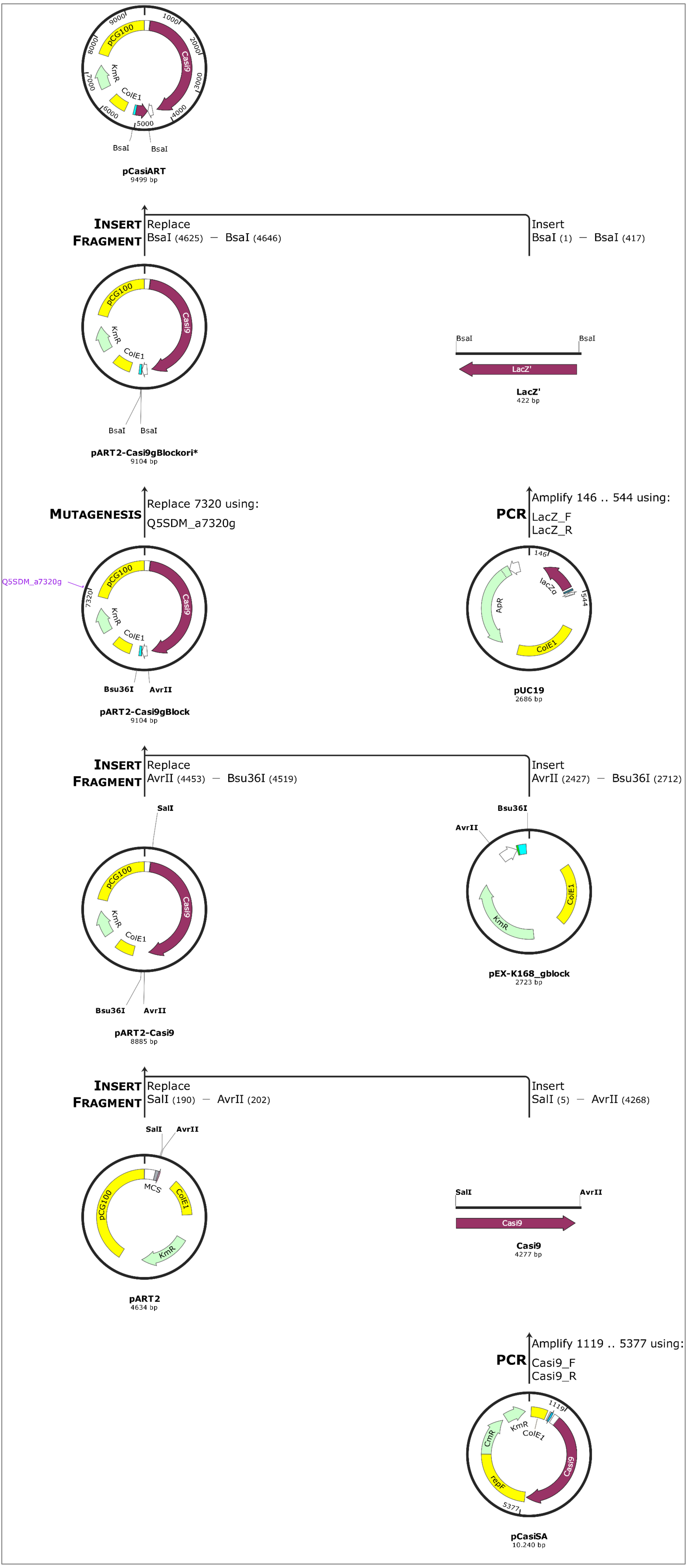

Fig. S1 Construction history of pCasiART with pART2 as the backbone. Created with SnapGene® software (Insightful Science; available at snapgene.com)

REFERENCES

1. Yanisch-Perron C, Vieira J, Messing J (1985) Improved M13 phage cloning vectors and host strains: nucleotide sequences of the M13mpl8 and pUC19 vectors. Gene 33:103–119. https://doi.org/10.1016/0378-1119(85)90120-9
2. Sandu C, Chiribau C-B, Sachelaru P et al. (2005) Plasmids for nicotine-dependent and -independent gene expression in *Arthrobacter nicotinovorans* and other *Arthrobacter* species. Appl Environ Microbiol 71:8920–8924. https://doi.org/10.1128/AEM.71.12.8920-8924.2005
3. Chen W, Zhang Y, Yeo W-S et al. (2017) Rapid and efficient genome editing in *Staphylococcus aureus* by using an engineered CRISPR/Cas9 system. J Am Chem Soc 139:3790–3795. https://doi.org/10.1021/jacs.6b13317
4. Koch C, Schumann P, Stackebrandt E (1995) Reclassification of *Micrococcus agilis* (Ali-Cohen 1889) to the genus *Arthrobacter* as *Arthrobacter agilis* comb. nov. and emendation of the genus *Arthrobacter*. Int J Syst Bacteriol 45:837–839. https://doi.org/10.1099/00207713-45-4-837
5. Flegler A, Runzheimer K, Kombeitz V et al. (2020) *Arthrobacter bussei* sp. nov., a pink-coloured organism isolated from cheese made of cow's milk. Int J Syst Evol Microbiol 70:3027–3036. https://doi.org/10.1099/ijsem.0.004125
